# Artificial Intelligence Approaches for Gastrointestinal Bleeding Detection in Capsule Endoscopy: A Systematic Review and Critical Analysis

**DOI:** 10.3390/diagnostics16213449

**Published:** 2026-10-22

**Authors:** Nasima Mohammad Helal, Jon Zaccary Regala, Mujeeb Rahman

**Affiliations:** Department of Biomedical Engineering, College of Engineering and Information Technology, Ajman University, Ajman P.O. Box 346, United Arab Emirates; nasimamdhelal@gmail.com (N.M.H.); 202310431@ajmanuni.ac.ae (J.Z.R.)

**Keywords:** artificial intelligence, machine learning, deep learning, gastrointestinal bleeding, small-bowel bleeding, wireless capsule endoscopy, video capsule endoscopy, risk of bias

## Abstract

**Background/Objectives:** Gastrointestinal (GI) bleeding is a common, potentially life-threatening condition, and capsule endoscopy (CE) is the principal investigation for suspected small-bowel bleeding after non-diagnostic upper endoscopy and colonoscopy. Because manual review of thousands of CE frames is labor-intensive, artificial intelligence (AI) has been proposed to automate bleeding detection. **Methods:** Following PRISMA 2020, this systematic review synthesizes 15 peer-reviewed journal studies (2020–2025) applying traditional machine learning, convolutional neural networks, transfer learning, attention-based or hybrid architectures, segmentation, or object detection to GI bleeding detection in CE. Methodological quality was appraised with an AI-adapted risk-of-bias framework informed by QUADAS-2 and PROBAST. **Results:** Frame-level classification accuracy ranged from 90.6% to 99.9% (median 98.5%; 13 studies), with the lowest values from evaluations closest to clinical practice; the segmentation and detection studies reported a mean intersection-over-union of 86.9% and an mAP@0.5 of 79.0%. Only two studies (13%) were at low risk of bias for validation strategy, none reported patient-level separation of training and test data, only four (27%) used clinical patient data, and none compared AI with conventional endoscopy or clinical outcomes. **Conclusions:** Current evidence demonstrates high performance on curated datasets but not yet generalizability or clinical readiness; patient-level, external, and prospective multicentre validation is required.

## 1. Introduction

Gastrointestinal (GI) bleeding refers to hemorrhage occurring anywhere along the GI tract, from the esophagus to the rectum [1]. Numerous pathological conditions may result in GI bleeding, including peptic ulcer disease, esophageal tears, vascular malformations, inflammatory disorders, and malignancies [2,3,4]. Among these, peptic ulcers remain one of the most common causes of upper GI bleeding [5,6]. Clinical manifestations range from hematemesis, melena, and hematochezia to occult blood loss with iron-deficiency anemia and nonspecific symptoms such as dizziness, syncope, fatigue, and abdominal pain.

The global burden of GI bleeding remains substantial. A recent systematic review reported annual incidence rates ranging from 15 to 172 cases per 100,000 population for upper GI bleeding and 20.5 to 87 cases per 100,000 population for lower GI bleeding, encompassing approximately 4.1 million reported patients between 1980 and 2012 [7]. More recent estimates indicate that upper GI bleeding affects approximately 80–150 individuals per 100,000 person-years and remains one of the leading GI conditions requiring hospital admission [8].

The diagnostic pathway depends on the suspected source of bleeding. Esophagogastroduodenoscopy (EGD), also termed oesophagogastroduodenoscopy (OGD) or upper endoscopy, is the first-line investigation for suspected upper GI bleeding and allows diagnosis and endoscopic hemostasis of common sources such as bleeding peptic ulcers, as well as diagnosis of gastric neoplasms [9]. Colonoscopy is the principal investigation for suspected lower GI bleeding [10], and computed tomography (CT) angiography and other radiological techniques assist in localizing active bleeding [11,12]. In approximately 5–10% of patients presenting with GI bleeding, however, no source is identified on upper endoscopy and colonoscopy, and the small bowel, which lies largely beyond the reach of standard flexible endoscopes, is then the most likely source [13]. In this setting of suspected small-bowel bleeding (previously termed obscure GI bleeding), capsule endoscopy (CE) is recommended as the first-line small-bowel investigation, enabling non-invasive visualization of the entire small intestine as a swallowed capsule captures tens of thousands of images [13,14,15]. CE is purely diagnostic: it does not allow biopsy or treatment, and lesions it identifies are subsequently confirmed and treated by device-assisted enteroscopy. CE therefore complements, rather than replaces, conventional flexible endoscopy.

Despite its diagnostic advantages, manual interpretation of CE examinations remains labor-intensive and susceptible to observer variability. Clinicians are often required to review tens of thousands of image frames for a single examination, which typically takes 30–120 min [16], increasing the likelihood of fatigue-related diagnostic errors and missed bleeding lesions. Consequently, automated computer-assisted diagnostic systems based on artificial intelligence have attracted increasing research interest [17].

Recent advances in artificial intelligence (AI) and machine learning (ML) have significantly enhanced automated image analysis in GI endoscopy. Deep learning (DL) architectures, particularly convolutional neural networks (CNNs), together with transformer-based networks, hybrid frameworks, and conventional supervised learning algorithms such as support vector machines (SVMs) and k-nearest neighbors (KNNs), have demonstrated promising performance for bleeding detection and GI disease classification [18,19,20,21]. These developments suggest considerable potential for improving diagnostic accuracy while reducing clinician workload.

Although a recent systematic review by Singh et al. [22] surveyed machine learning methodologies for video capsule endoscopy (VCE)-based bleeding analysis, its scope encompassed studies in which bleeding was investigated alongside other GI abnormalities, including polyps, ulcers, inflammatory lesions, and other pathological findings. Consequently, the review provided a broad overview of AI applications for bleeding analysis in capsule endoscopy rather than an in-depth synthesis focused exclusively on GI bleeding detection. With 114 studies spanning 2008–2024, it offers a valuable map of the wider field, and its quality assessment applied the generic QUADAS-2 and PROBAST tools to 104 of the included studies.

In contrast, the present systematic review focuses exclusively on AI methods developed for GI bleeding detection in CE, including classification, localization, segmentation, and object detection. By restricting the inclusion criteria to studies in which GI bleeding constituted the primary research objective and incorporating the most recent evidence published between 2020 and 2025, this review provides a focused and up-to-date synthesis of the field, intended as a concise, dedicated reference for readers who specifically need to know how AI performs for GI bleeding detection in CE, how strong the supporting evidence is, and what is still required before clinical use. As summarized in Table 1, 12 of the 15 studies included in the present review were also included by Singh et al. [22], whereas the remaining three were not; because the two reviews draw on much of the same primary literature, the contribution of the present review lies not in identifying different studies but in its dedicated bleeding-specific perspective, an AI-specific methodological quality assessment that explicitly addresses data leakage and patient-level separation, critical analysis of current challenges, research gaps, and future directions, and the inclusion of the most recent evidence. In addition to comparing AI methodologies, datasets, reference standards, validation strategies, and reported diagnostic performance, this review critically appraises the methodological rigor of the included studies using an AI-adapted risk-of-bias framework informed by QUADAS-2 and PROBAST principles [23,24]. Furthermore, it identifies key barriers to clinical translation and provides evidence-based recommendations to guide the development of more robust, reproducible, and clinically applicable AI-assisted capsule endoscopy systems.

## 2. Materials and Methods

### 2.1. Review Design

This systematic review was conducted to identify, evaluate, and synthesize studies applying ML and DL techniques for GI bleeding detection in CE. The review considered studies addressing bleeding classification, localization, segmentation, and detection using CE image or video data. The review was conducted and reported in accordance with the Preferred Reporting Items for Systematic Reviews and Meta-Analyses (PRISMA) 2020 statement [38]; the completed PRISMA 2020 checklist is provided as Supplementary Table S2.

Studies published between January 2020 and December 2025 were considered for inclusion, reflecting recent advances in AI-based medical image analysis and CE interpretation. The objective of this review was to provide a comprehensive overview of ML- and DL-based approaches for GI bleeding detection in CE, compare their methodological characteristics and reported performance, assess methodological quality, and identify current limitations and future research directions.

The review was not registered in a systematic review registry (e.g., PROSPERO), and no prospectively registered or published review protocol was available. Accordingly, amendments to a registered protocol were not applicable.

### 2.2. Information Sources

A comprehensive literature search was conducted across Google Scholar, PubMed, Scopus, IEEE Xplore, ScienceDirect, and MDPI to provide broad coverage of biomedical, clinical, engineering, and computer science literature relevant to AI applications in CE. Reference lists of relevant articles were additionally examined through citation searching to identify potentially eligible studies that may not have been retrieved through the primary electronic searches.

Literature identification began in 2024. Google Scholar, PubMed, Scopus, IEEE Xplore, ScienceDirect, and MDPI were each last searched in June 2026. Only studies published between January 2020 and December 2025 were eligible for inclusion.

### 2.3. Literature Search Strategy

The search strategy combined keywords related to GI bleeding, CE, and AI. The search query was iteratively refined by testing different keyword combinations and evaluating the relevance of the retrieved studies. This process enabled the development of a bleeding-focused search strategy while maintaining broad coverage of ML and DL approaches in CE.

The principal Boolean search strategy was applied across all six sources, with minor syntax adaptations where necessary to accommodate differences in database search interfaces; the core concepts and terms were retained across all sources. Because the database-specific search histories were not prospectively archived, the exact source-specific syntax could not be reconstructed retrospectively; therefore, the common search strategy is reported here. The principal Boolean search expression was formulated as follows:

(“gastrointestinal bleeding” OR “GI bleeding” OR “intestinal bleeding” OR “hemorrhage”)

AND

(“capsule endoscopy” OR “wireless capsule endoscopy” OR “video capsule endoscopy”)

AND

(“machine learning” OR “deep learning” OR “CNN” OR “SVM” OR “transformer” OR “U-Net”)

The search was restricted to English-language studies published between 2020 and 2025. Reference-list and citation searching supplemented the electronic searches. For the purposes of this review, the term CE is used as a general term encompassing wireless capsule endoscopy (WCE) and VCE, which were considered equivalent during the literature search and study selection.

### 2.4. Eligibility Criteria

Original peer-reviewed journal articles published between January 2020 and December 2025 were eligible when GI bleeding represented a primary focus of the investigation and ML or DL methods were applied to bleeding classification, detection, localization, or segmentation using CE data. Publication date was determined from the date of first online publication. Studies were required to provide sufficient information regarding dataset characteristics, methodological approach, validation strategy, and quantitative performance to permit meaningful assessment and comparison.

Studies were excluded if they focused primarily on GI abnormalities other than bleeding, did not use CE data, did not employ an AI-based approach, relied solely on conventional image-processing or statistical methods, or were published before 2020. Review articles, surveys, editorials, book chapters, theses, conference papers and abstracts, posters, patents, and preprints were also excluded. Papers published in conference proceedings series, including proceedings issued under journal-style titles (e.g., *Procedia Computer Science*), were classified as conference papers. Only English-language articles were considered. Reports for which the full text could not be retrieved were recorded as not retrieved and were not subjected to full-text eligibility assessment.

The inclusion and exclusion criteria applied throughout the screening process are summarized in Table 2.

### 2.5. Selection Process

All identified records were consolidated into a common screening pool, and duplicate records were removed before eligibility screening. Study selection was performed by one reviewer (N.M.H.), and all screening decisions were checked by a second reviewer (J.Z.R.). Screening involved keyword and title assessment, followed by abstract screening and full-text eligibility assessment according to the predefined inclusion and exclusion criteria. Discrepancies between the two reviewers were resolved through discussion and consensus. No automated screening tools were used.

A total of 218 records were identified, comprising 210 records identified through database searching and eight records identified through citation searching. After removal of 76 duplicate records, 142 unique records remained for keyword and title screening. A total of 101 records were excluded at this stage, leaving 41 abstracts for eligibility assessment. Following abstract screening, 15 records were excluded, including five published before 2020 and ten that did not satisfy the eligibility criteria. Consequently, 26 reports were sought for full-text retrieval.

**Figure 1:**
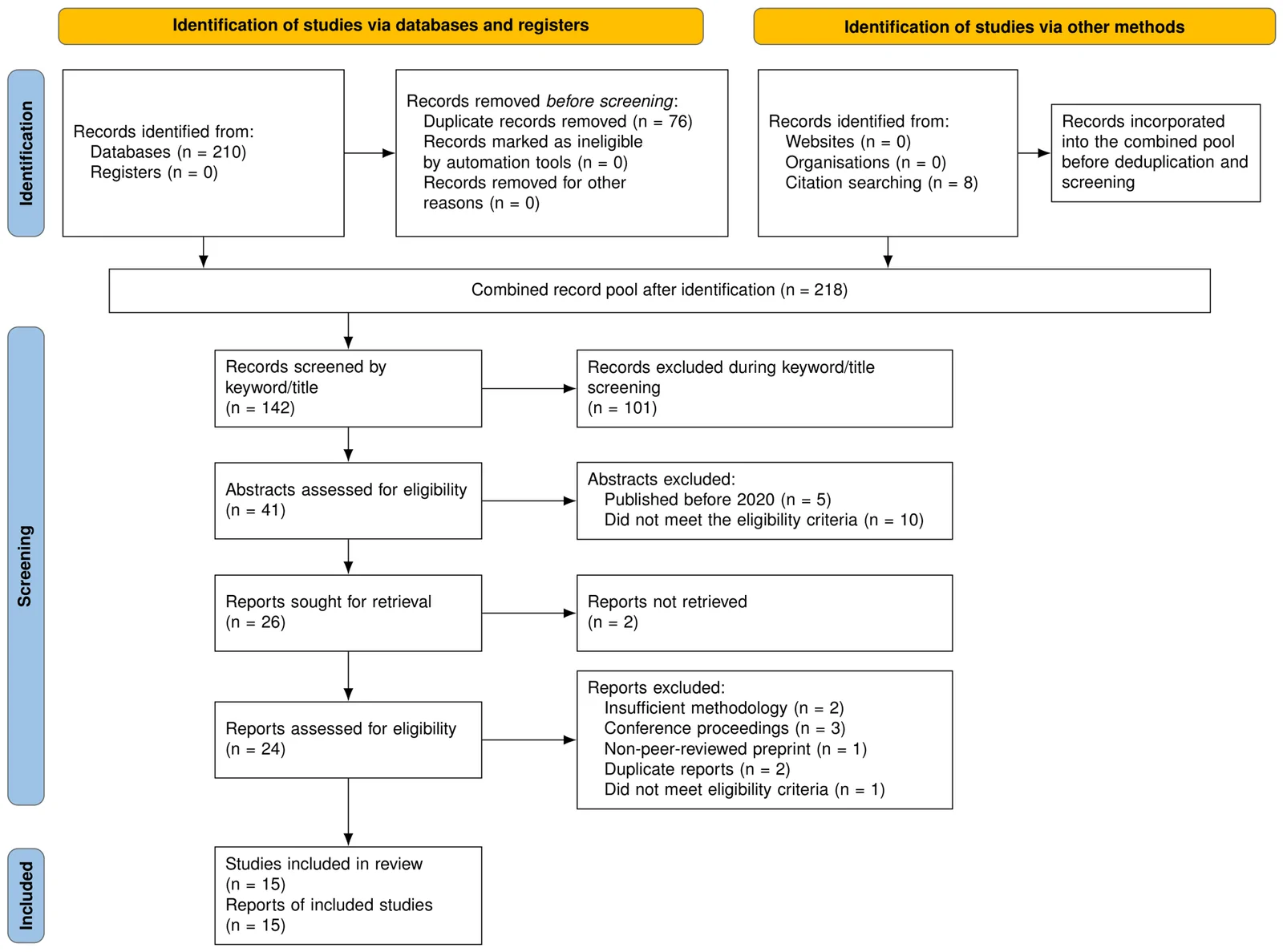
PRISMA 2020 flow diagram illustrating the identification, screening, eligibility assessment, and inclusion of studies in the systematic review.

Two reports could not be retrieved, leaving 24 reports for full-text eligibility assessment. Nine reports were excluded following full-text assessment: two because of insufficient methodological reporting, three because they were published in conference proceedings, one because it was a preprint that had not been peer-reviewed, two because they represented duplicate reports of studies already considered, and one because it did not meet the predefined eligibility criteria. Consequently, 15 studies were included in the qualitative synthesis. Figure 1 presents the PRISMA 2020 flow diagram, and the reports excluded at full-text assessment are listed with reasons in Supplementary Table S1.

### 2.6. Data Collection Process and Data Items

Data extraction was performed by one reviewer (N.M.H.), and all extracted data were checked by a second reviewer (J.Z.R.) against the original publications to improve consistency and minimize transcription or interpretation errors. Discrepancies were resolved through discussion and re-examination of the corresponding publication. No automated data-extraction tools were used.

For each included study, information was extracted regarding publication characteristics, methodological approach, learning paradigm, task type (classification, localization, segmentation, or detection), dataset characteristics, public availability of the dataset, sample size, image or video modality, reference standard (source of ground-truth labels), data augmentation techniques, validation strategy, computational resources (when reported), quantitative performance metrics, and the principal findings, reported limitations, and proposed future research directions. Reported performance metrics included accuracy, specificity, precision, recall (sensitivity), F1-score, negative predictive value (NPV), area under the receiver operating characteristic curve (AUC), Dice coefficient, Intersection over Union (IoU), global accuracy, mean Average Precision (mAP), and other evaluation measures where applicable. Metrics not reported by an individual study were recorded as not reported (NR), and missing values were not estimated or imputed. Where a study reported several model configurations or datasets, the configuration designated by the authors as the proposed method on the primary dataset was used as the headline value; additional results (e.g., on a second or independent dataset) are reported in Section 3.2.

### 2.7. Methodological Quality and Risk-of-Bias Assessment

To evaluate the methodological quality and potential risk of bias of the included studies, an AI-adapted methodological quality assessment framework was developed based on concepts derived from the QUADAS-2 and PROBAST tools [23,24]. As the included studies primarily investigated AI models for GI bleeding detection in CE rather than conventional diagnostic accuracy or prognostic prediction models, selected domains from these established frameworks were adapted to assess methodological aspects specific to AI-based medical image analysis. At the time the assessment was designed (2024), the AI-specific extension of QUADAS (QUADAS-AI) had been announced but was not yet available as a finalized instrument [39], and PROBAST+AI, which was published in 2025, is designed for prediction-model studies rather than image-level algorithm development studies [40]. The proposed assessment framework is presented in Table 3.

The assessment was performed by one reviewer (N.M.H.), and all ratings were verified by a second reviewer (J.Z.R.) against the original publications, with disagreements resolved through discussion and consensus. Six domains were defined, together with their rating criteria, before the assessment began: dataset representativeness, reference standard, validation strategy, data handling, reporting transparency, and clinical applicability. No automated tools were used. Dataset representativeness evaluated whether the study population and dataset reflected clinically representative CE examinations. The reference standard assessed whether bleeding annotations or ground truth labels were established by expert clinicians or validated public datasets. Validation strategy examined the use of patient-level splitting, cross-validation, independent testing, or external validation. Data handling evaluated the reporting of preprocessing procedures, data augmentation, class imbalance management, and data partitioning. Reporting transparency assessed the completeness of methodological and experimental reporting, including model architecture, training procedures, and evaluation metrics. Clinical applicability evaluated the potential for translation into routine clinical practice and real-world CE interpretation.

#### Risk Rating Criteria

To improve consistency and transparency, predefined criteria were established for assigning low-, high-, and unclear-risk judgments within each methodological domain. Dataset representativeness was considered low risk when studies used clinically representative datasets with clearly described patient populations, an adequate sample size, or validated public CE datasets. Studies based on very small, highly selected, or synthetic datasets were classified as high risk, whereas insufficiently described datasets were rated as unclear.

Reference standard was considered low risk when bleeding labels, segmentation masks, or annotations were established by expert clinicians or validated public datasets. High risk was assigned when the ground truth was poorly defined, unreliable, or lacked clinical verification, while studies that did not adequately describe the annotation process were classified as unclear.

Validation strategy was rated as low risk when external validation, cross-dataset validation, or patient-level validation was performed. Studies relying solely on simple train–test splits were considered high risk because such approaches may overestimate model performance, whereas studies using cross-validation without reporting patient-level separation or those with insufficiently described validation procedures were classified as unclear. Each study was rated on the most robust validation strategy it reported for CE data: a study reporting both a random split and k-fold cross-validation without patient-level separation was therefore rated unclear. An additional test set qualified as external validation only when it consisted of CE images from an identified independent source and quantitative results were reported; test sets of unverifiable provenance (e.g., images collected through internet search engines) and test sets of non-capsule (conventional endoscopy) images did not qualify.

Data handling was considered low risk when preprocessing, data augmentation, class imbalance management, and data-splitting procedures were clearly described and appropriately implemented. High-risk ratings were assigned when important data handling issues were ignored or inappropriate preprocessing was evident, whereas insufficient reporting resulted in an unclear-risk judgment. Applying data augmentation to the full dataset before partitioning it into training and test sets (or before cross-validation) was considered inappropriate, because augmented copies of the same source image may then appear in both training and test data.

Reporting transparency was rated as low risk when sufficient methodological details, including dataset characteristics, model architecture, training procedures, and evaluation metrics, were provided to enable reproducibility. Studies with major methodological omissions were considered high risk, while partially reported methodologies were classified as unclear.

Finally, clinical applicability was considered low risk when the proposed AI system was evaluated using real clinical data, defined as CE examinations from an identified clinical patient cohort or real clinical CE video. Discussion of potential workflow integration without such an evaluation was not considered sufficient for a low-risk rating. Studies representing only proof-of-concept experiments with unrealistic evaluation settings, such as evaluation confined to a very small image set expanded mainly by augmentation, were classified as high risk, whereas studies evaluated only on public benchmark images or providing only limited evidence regarding clinical applicability were rated as unclear.

Each domain was independently assessed and classified as *low risk*, *high risk*, or *unclear risk* based on the methodological information reported in each study. A *low-risk* rating indicated that the criterion was adequately addressed and sufficiently described. A *high-risk* rating indicated methodological limitations that could introduce bias or reduce the reliability of the reported findings. An *unclear-risk* rating was assigned when insufficient methodological information was available to confidently determine the quality of the study. During revision, all ratings were re-assessed against the original publications using the operational rules above, and the revised ratings are reported in Section 3.10; each rating that changed from the initial assessment is listed with its justification in Supplementary Table S3.

### 2.8. Data Synthesis and Analysis

A qualitative narrative synthesis was performed because substantial methodological and clinical heterogeneity among the included studies precluded a meaningful quantitative meta-analysis. Studies differed in dataset source and composition, sample size, task definition, AI architecture, validation strategy, class distribution, and reported performance measures. Pooling performance estimates across these heterogeneous experimental settings could therefore have produced misleading summary estimates.

Studies were grouped according to their principal methodological approach and diagnostic task, namely traditional ML, CNN-based DL trained end-to-end, transfer learning and feature fusion with pretrained backbones, segmentation networks, attention-based or hybrid architectures, and object detection methods. Each study was assigned to one primary category, which is used consistently in Table 4, in the comparative synthesis (Section 4.3), and in the narrative synthesis. Reported numerical performance values were retained as presented in the original publications. Potential sources of heterogeneity were explored descriptively. No subgroup meta-analysis or meta-regression was conducted because no pooled quantitative synthesis was undertaken; the effect of excluding one study published in a journal whose indexing status changed was checked descriptively (Section 4.8).

Formal statistical assessment of publication bias, such as funnel-plot analysis, was not performed because no meta-analysis was conducted; potential reporting limitations were considered qualitatively through the reporting-transparency domain. A formal certainty-of-evidence framework such as GRADE was not applied because the review synthesized heterogeneous AI development studies rather than estimating a common clinical effect; the strength and applicability of the evidence were instead considered through the methodological quality assessment and critical qualitative synthesis.

## 3. Results

### 3.1. Study Selection

The study-selection process is summarized in Figure 1. Of 218 records identified (210 from database searching and eight from citation searching), 76 duplicates were removed and 142 records underwent keyword and title screening, of which 101 were excluded. The remaining 41 records underwent abstract screening, resulting in the exclusion of 15 records. Full-text retrieval was sought for 26 reports; two could not be retrieved, leaving 24 reports for full-text eligibility assessment. Nine reports were excluded at this stage (Supplementary Table S1), and 15 studies were included in the qualitative synthesis.

### 3.2. Overall Study Characteristics

Fifteen studies met the predefined eligibility criteria and were included in the qualitative synthesis. To facilitate comparison, the included studies were categorized according to their primary AI methodology, namely traditional ML (*n* = 3), CNN-based DL trained end-to-end (*n* = 3), transfer learning and feature fusion (*n* = 5), segmentation networks (*n* = 1), hybrid or attention-based architectures (*n* = 2), and object detection methods (*n* = 1). Thirteen studies addressed frame-level classification (two of which also performed segmentation or localization), one addressed pixel-level segmentation, and one addressed object detection. Reported classification accuracy ranged from 90.6% to 99.9% (median 98.5%). The methodological characteristics, datasets, validation strategies, quantitative performance, and key findings of the included studies are summarized in Table 4 and Table 5. The principal findings for each methodological category are presented below.

### 3.3. Reference Standards Used in the Included Studies

In all included studies, the reference standard was a frame-level (or pixel-level) label assigned to CE images by human readers, either directly by the study team or through a public dataset. Labels were established by expert clinicians within the study in six studies: two gastroenterologists with consensus [16], two capsule experts with confirmation by a third [35], gastroenterologists [25,27], expert physicians [31], or a professional endoscopist [33]. Five studies relied on the labels supplied with public datasets, such as KID, MICCAI 2017, Kvasir-Capsule, and Auto-WCEBleedGen, whose annotations were produced or verified by clinical experts [18,29,30,32,36]. In four studies, the source of the bleeding labels or the annotation process was not described, including the Kaggle dataset used by HARU-Net [26,28,34,37]. No included study used clinical outcomes, endoscopic or surgical confirmation of the bleeding source, or a direct comparison with conventional flexible endoscopy as the reference standard. Consequently, the reported metrics reflect agreement between the algorithm and expert CE image reading, not the diagnostic accuracy of CE itself for identifying or localizing bleeding sources.

### 3.4. Traditional Machine Learning Approaches

Traditional ML approaches primarily relied on handcrafted colour and texture feature extraction combined with conventional classifiers such as SVMs and KNNs. Despite their comparatively simpler architectures, these methods achieved competitive performance, demonstrating that carefully engineered feature descriptors remain effective for GI bleeding detection.

Al Mamun et al. [26] proposed a bleeding recognition framework combining fuzzy logic-based edge detection, principal component analysis (PCA), and a quadratic SVM classifier. Using 10-fold cross-validation on 2393 annotated WCE images, the proposed method achieved an accuracy of 98.2%, an F1-score of 95.4%, and a negative predictive value of 99%, outperforming several conventional classifiers, including KNN. The authors reported strong performance for handcrafted feature engineering; however, the framework remained dependent on image pre-processing and was evaluated on a single dataset without external validation.

Rathnamala and Jenicka [32] developed a classical SVM classifier using Gaussian mixture model-based superpixel segmentation and colour-texture feature extraction. The proposed framework achieved one of the highest performances among traditional ML methods, with an accuracy of 99.88%, an F1-score of 99.91%, and an AUC of 1.00 on a 50:50 image-level split, while also providing bleeding region localization through image segmentation. The authors demonstrated that carefully designed handcrafted features remain highly competitive for bleeding detection, although they recommended incorporating richer colour representations to improve discrimination of subtle bleeding variations.

Vajravelu et al. [34] proposed a dual-stage framework for bleeding frame classification and bleeding region localization using colour feature extraction, region-of-interest analysis, and conventional ML classifiers. The method achieved an accuracy of 98.86% and an AUC of 0.9771, although the abstract of the same report states an accuracy of 95.75%, while saliency map generation improved visualization of suspected bleeding regions. The source of the 30 publicly available videos was not specified. Nevertheless, broader evaluation on larger and more heterogeneous datasets would better establish the model’s generalizability across different clinical settings.

Overall, traditional ML approaches demonstrated that handcrafted feature engineering remains capable of achieving high classification performance. However, these methods generally relied on manually designed colour and texture descriptors, limiting their adaptability to variations in illumination, capsule devices, and bleeding appearance when compared with modern deep learning approaches.

### 3.5. CNN-Based Deep Learning Approaches

This category comprises CNNs trained end-to-end on CE images without a pretrained backbone; studies using pretrained networks are described in Section 3.6.

Pannu et al. [25] proposed a supervised CNN ensemble for bleeding classification. The incorporation of image augmentation techniques, including rotation and noise injection, enhanced model robustness to image variability commonly encountered in clinical practice. The proposed framework achieved an accuracy of 95% and an F1-score of 92% on a public dataset. The trained model was further tested, without retraining, on 5000 frames from a real clinical CE video annotated by a gastroenterologist, achieving an accuracy of 93%, which supports the feasibility of applying CNN-based approaches beyond the development dataset. The authors recommended expanding video datasets and developing automated annotation and segmentation systems to support future clinical implementation.

Latha et al. [28] developed a CNN-based classifier for intestinal hemorrhage detection, achieving an accuracy of 95.7%. The authors investigated the influence of batch size and training epochs through a limited ablation study. However, the dataset description was incomplete: the study reports 4778 “Kvasir” images together with approximately 1500 frames from a private 30-min WCE video, without explaining how the latter were used. Future work was directed toward extending the framework to real-time clinical applications.

Farej et al. [30] proposed a lightweight CNN trained on 892 Kvasir-Capsule images and implemented it on a Raspberry Pi 4 platform, highlighting the feasibility of resource-efficient deployment. The model achieved an accuracy of 99.4% with an 80:20 split (98.5% with a 70:30 split) and correctly classified all 56 frames of a short test video from a different capsule system. The authors recommended expanding the dataset and evaluating the framework under more diverse imaging conditions.

### 3.6. Transfer Learning and Feature Fusion Approaches

Transfer learning approaches sought to improve bleeding detection by leveraging pretrained convolutional neural networks, either fine-tuned end-to-end or used as feature extractors combined with conventional machine learning classifiers or additional segmentation frameworks.

Caroppo et al. [18] proposed an expert system integrating three pretrained CNN architectures, namely VGG19, InceptionV3, and ResNet50, for feature extraction, followed by conventional machine learning classifiers. Among the evaluated classifiers, SVM achieved the best overall performance, reaching an accuracy of 95.70% on the MICCAI dataset and 98.20% on the KID dataset. When trained on MICCAI 2017 and tested on KID, accuracy was 93.41%, the only cross-dataset evaluation among the included studies. Because augmentation was applied before cross-validation, the internal cross-validation estimates may be optimistic. The authors identified dataset expansion, exploration of additional deep learning architectures, and broader clinical validation as important directions for future work.

Ghosh and Chakareski [31] combined transfer learning using AlexNet with semantic segmentation (SegNet) to simultaneously classify bleeding frames and localize bleeding regions. The proposed framework achieved an F1-score of 98.49% for bleeding frame classification using random 60:40 image-level splits of 2350 frames from 32 videos (83.24% accuracy on KID), together with a global segmentation accuracy of 94.42%. The authors reported better performance than alternative transfer learning architectures, colour spaces, and previously published methods. The integration of segmentation further enhanced clinical interpretability by providing explicit localization of bleeding areas. Future work focused on expanding available datasets and developing a complete computer-aided detection system for capsule endoscopy videos.

Rustam et al. [27] combined MobileNet-based transfer learning with a customized CNN classifier, incorporating image flipping as a data augmentation strategy. Using 5000 images from 33 patients labelled by gastroenterologists, the proposed hybrid architecture achieved an accuracy of 99.3% and an F1-score of 99.7% on a 150-image test set, and 97.8% on an additional internet-collected image set, representing one of the highest-performing classification models included in this review. The authors reported better performance than conventional machine learning techniques and previously published deep learning models. The authors recommended further dataset expansion and external clinical validation to improve model generalizability.

Saraiva et al. [16] fine-tuned an ImageNet-pretrained Xception network on 22,095 frames extracted from 1483 CE examinations performed at a single tertiary centre, labelled by two gastroenterologists with consensus. Using a random 80:20 image-level split, the model achieved an accuracy of 98.5%, a sensitivity of 98.6%, and a specificity of 98.9%, and processed approximately 184 frames per second. The authors acknowledged image-level rather than patient-level analysis, evaluation on selected still frames rather than full-length videos, use of a single capsule system, and the absence of an assessment of reading-time reduction.

Turck et al. [35] validated a transfer learning framework based on InceptionV3 using a real-world clinical capsule endoscopy dataset. Although the model achieved an accuracy of 90.6%, the study intentionally evaluated performance under realistic clinical conditions rather than highly curated benchmark datasets. The authors identified limited dataset size and the potential for data leakage arising from patient-level overlap between training and validation sets as important methodological limitations. Future studies should investigate larger multicentre datasets and compare transfer learning architectures under standardized evaluation protocols.

### 3.7. Segmentation Approaches

Unlike conventional classification models that determine the presence or absence of bleeding, segmentation approaches provide pixel-level localization of bleeding regions, thereby improving interpretability and supporting clinical decision-making.

Li et al. [33] introduced DFCA-Net, a dual feature context aggregation network that combines ResNet and Res2Net backbones to separately extract colour and texture information before feature fusion. The model achieved an mIoU of 86.9% and a pixel accuracy of 91.3%. In comparisons with other segmentation methods and ablation experiments, the authors reported improved performance, particularly for small bleeding regions and regions affected by image artefacts such as bubbles. Nevertheless, the authors acknowledged that further evaluation on larger clinical datasets would strengthen the evidence for clinical applicability.

### 3.8. Attention-Based and Hybrid Architectures

Lafraxo et al. [29] proposed a hybrid architecture in which a regularized CNN extracts spatial features that are then classified by a gated recurrent unit (GRU) network, and compared it with CNN, CNN–RNN, and CNN–BiLSTM variants on 3295 images from the MICCAI 2017 dataset without data augmentation. The CNN–GRU model achieved the best performance, with an accuracy of 99.39%, a sensitivity of 99.09%, a specificity of 99.54%, and an AUC of 1.00 on a 60/20/20 hold-out split, and a mean accuracy of 98.33% under 5-fold cross-validation. Because the GRU operated on features of individual, non-sequential images, the recurrent component modelled feature dependencies rather than temporal information across consecutive video frames. The authors proposed extending the approach to localization, segmentation, and additional datasets.

Balasubaramanian et al. [37] proposed HARU-Net, an attention-enhanced residual U-Net that combines histogram equalization with gated attention mechanisms for automated bleeding classification. The lightweight architecture achieved a mean classification accuracy of 98.21% under 5-fold cross-validation, outperforming both the conventional Attention U-Net and several transfer learning models. However, the dataset comprised only 226 original images, each augmented approximately 21-fold before partitioning. The authors recommended future investigation of alternative activation functions, optimization strategies, larger multicentre datasets, and additional explainability techniques.

### 3.9. Object Detection

Chai and Zhang [36] introduced DMP-WCEBleedNet, a YOLOv11-based object detection framework incorporating a poly kernel inception block, self-modulated feature aggregation, and a dynamic detection head. Extensive ablation experiments demonstrated that each architectural component contributed to improved lesion detection performance. In addition to classification, the framework provides explicit localization of bleeding regions, offering valuable visual support for clinicians. Although the model achieved the highest reported detection performance on the Auto-WCEBleedGen benchmark (mAP@0.5 of 79.0%), the data partitioning was not reported, and further validation on independent clinical datasets is required to establish generalizability.

Overall, the reviewed studies demonstrate a clear evolution from conventional handcrafted feature engineering toward increasingly sophisticated deep learning frameworks capable of classification, localization, and segmentation. Despite the consistently high diagnostic performance reported across many studies, recurrent limitations include restricted dataset diversity, limited external and multicentre validation, reliance on image-level rather than patient-level analysis, and the need for improved interpretability and clinical workflow integration.

### 3.10. Methodological Quality Assessment Results

**Figure 2:**
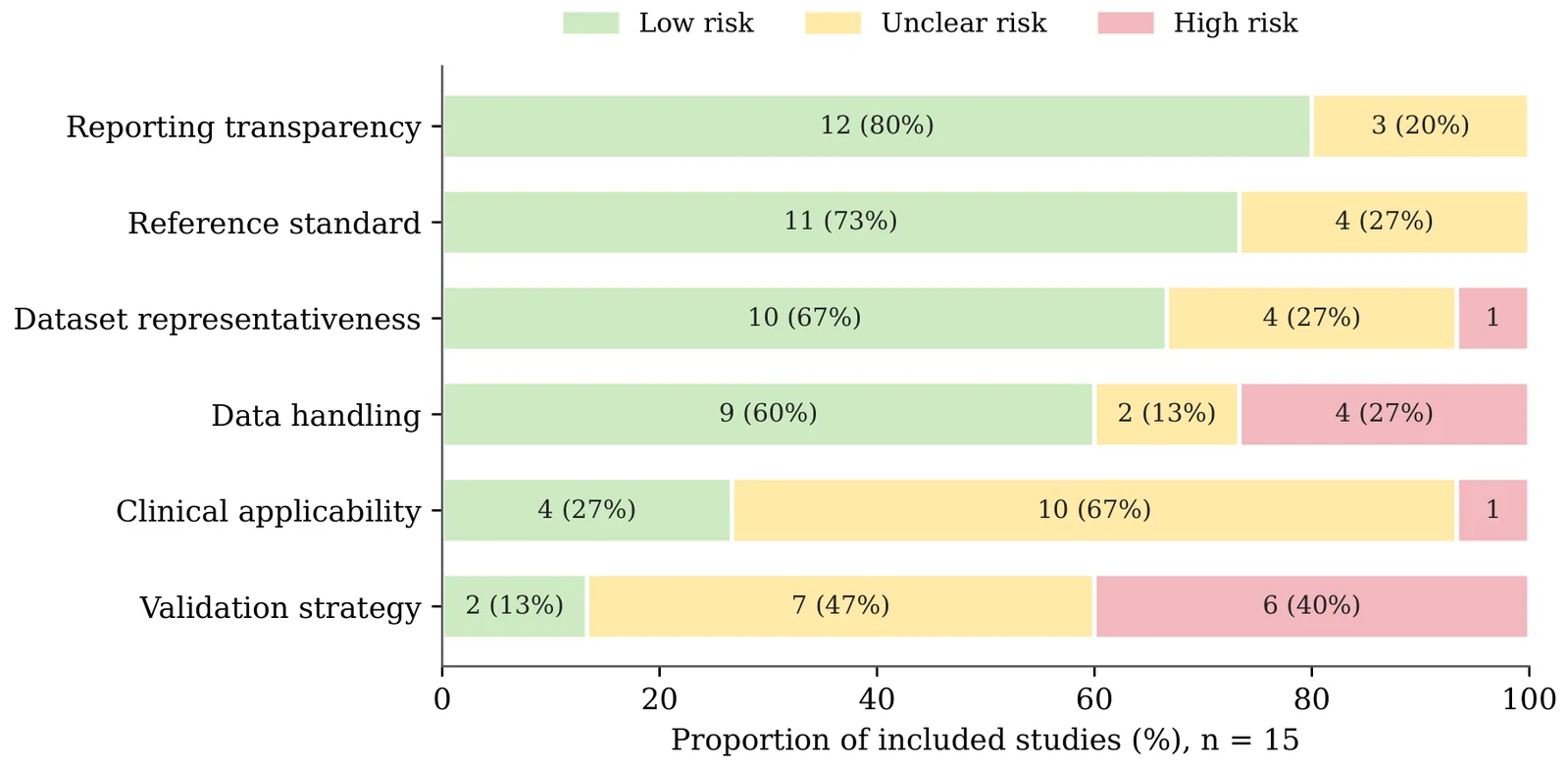
Summary of the methodological quality assessment across the included studies (*n* = 15). Horizontal stacked bars illustrate the proportion of studies classified as low, unclear, or high risk within each methodological domain according to the proposed AI-adapted risk-of-bias framework.

The methodological quality of the included studies was assessed using the proposed AI-adapted risk-of-bias framework described in Section 2.7. The individual study assessments are presented in Table 6, while the overall distribution of methodological quality across the evaluated domains is summarized in Figure 2.

Reporting transparency was the strongest domain, with 12 studies (80.0%) at low risk and three (20.0%) unclear. The reference standard was at low risk in 11 studies (73.3%) and unclear in four (26.7%) in which the annotation process was not described. Dataset representativeness was at low risk in ten studies (66.7%), unclear in four (26.7%), and high risk in one (6.7%) based on 226 original images.

Data handling discriminated more clearly between studies than in the initial assessment: nine studies (60.0%) were at low risk, two (13.3%) were unclear because data partitioning was not adequately reported, and four (26.7%) were at high risk because data augmentation was applied before partitioning or cross-validation, so that augmented copies of the same image could appear in both training and test data [18,27,33,37].

Validation strategy remained the weakest domain. Only two studies (13.3%) were at low risk: one tested its model on an independent, gastroenterologist-annotated clinical video [25] and one performed cross-dataset testing [18]. Seven studies (46.7%) were rated unclear because they relied on cross-validation without patient-level separation or did not report data partitioning, and six (40.0%) were at high risk because performance was estimated on a random image- or finding-level split. No included study reported patient-level separation of training and test data.

For clinical applicability, four studies (26.7%) were at low risk because their models were evaluated on CE data from clinical patient examinations [16,25,27,35], ten (66.7%) were unclear because evaluation was limited to public benchmark images, and one (6.7%) was at high risk because evaluation was confined to a very small, heavily augmented image set. No study evaluated an AI system prospectively or within a clinical reading workflow.

## 4. Discussion

### 4.1. Principal Findings

This systematic review identified 15 peer-reviewed journal studies, published between 2020 and 2025, that applied AI to GI bleeding detection in CE. Three findings stand out. First, reported performance was uniformly high: frame-level classification accuracy ranged from 90.6% to 99.9% (median 98.5%), irrespective of whether handcrafted features, end-to-end CNNs, transfer learning, or attention-based architectures were used. Second, the evidence supporting these figures is methodologically weak. No study separated training and test data at the patient level, only two performed any form of independent or cross-dataset testing, four applied augmentation in a way that allows near-duplicate images to appear in both training and test data, and only four evaluated their models on data from clinical patient examinations. Third, the lowest accuracies were reported by the studies whose evaluation was closest to clinical practice: 90.6% on real-world clinical data from a single centre [35], 93% on an independent clinical video [25], and 93.4% under cross-dataset testing [18]. Taken together, current evidence establishes the technical feasibility of AI-assisted bleeding detection on curated CE images, but it does not yet establish generalizability to unseen patients, devices, or centres, nor clinical benefit.

### 4.2. Evolution of AI Approaches for Gastrointestinal Bleeding Detection

The included studies show a methodological progression from handcrafted colour and texture features with conventional classifiers [26,32,34] to end-to-end CNNs [25,28,30] and, most commonly, transfer learning with ImageNet-pretrained backbones [16,18,27,31,35], which allows deep models to be trained on relatively small annotated CE datasets. Handcrafted approaches remained competitive because active bleeding has a distinctive chromatic signature, but their dependence on predefined colour descriptors limits robustness to differences in illumination and capsule hardware. More recent work has moved beyond frame-level classification towards pixel-level segmentation [31,33], attention-based and hybrid architectures [29,37], and single-stage object detection [36], which provide localization outputs that are more interpretable for readers. These localization tasks, however, require pixel- or box-level annotation and are represented by only one study each in the eligible journal literature.

### 4.3. Comparative Analysis of AI Models and Performance

Table 7 summarizes the six AI categories. Accuracy ranges overlapped substantially across categories (traditional ML 98.2–99.9%; end-to-end CNNs 95.0–99.4%; transfer learning 90.6–99.4%; attention/hybrid 98.2–99.4%), and the differences between categories are smaller than the differences attributable to evaluation design. The highest values were obtained on small or curated public datasets with random image-level splits or cross-validation, whereas the lowest values in the transfer-learning category came from the studies using real clinical data [35] or a second public dataset [31]. Within the attention/hybrid category, the 98.2% reported for HARU-Net was obtained on 226 original images expanded about 21-fold before partitioning [37], and the recurrent component of the CNN–GRU model operated on non-sequential images rather than video sequences [29]. Segmentation and detection metrics (mIoU 86.9% [33]; mAP@0.5 79.0% [36]) are not comparable with classification accuracy and each rests on a single study.

Consequently, the available evidence does not allow any AI approach to be identified as superior. CNN-based and transfer-learning approaches are the most frequently investigated and often report high internal performance, but no approach has yet been consistently validated on independent patients or external datasets. Apparent differences between approaches are more likely to reflect differences in datasets, class balance, augmentation, and validation design than true methodological superiority.

### 4.4. Validation, Data Leakage, and Clinical Applicability

The quality assessment (Table 6, Figure 2) indicates that the main threats to the validity of the reported performance arise from how test data were constructed. CE examinations produce long sequences of highly similar consecutive frames, and a single lesion is typically captured in many of them. When frames are randomly allocated to training and test sets, or when augmented copies of the same image are distributed across folds, the test set is not independent of the training set, and performance is likely to be overestimated. This risk was explicitly acknowledged by Turck et al., whose random finding-level split allowed images from the same patients to appear in both sets [35], and it applies to most included studies. Patient-level partitioning, external testing on data from other centres and capsule systems, and reporting of per-patient as well as per-frame results are therefore prerequisites for credible performance estimates.

Dataset representativeness is a related concern. Several studies used small, class-balanced, or heavily augmented image collections; augmentation increases geometric and photometric variability but cannot reproduce the biological variability across patients, bleeding patterns, bowel preparation, and capsule devices [33,37]. Two widely used public sources illustrate a further problem: the Auto-WCEBleedGen dataset was compiled from multiple internet resources [36], and the Kaggle dataset used by HARU-Net contains 226 original images [37], so that patient provenance and the possibility of overlap between training and test material cannot be verified.

Finally, although four studies evaluated their models on data from clinical patient examinations [16,25,27,35], none was prospective, none analysed complete examinations within a reading workflow, and none measured reading time, diagnostic yield, or clinical decision-making. The clinical value of these systems therefore remains to be demonstrated.

### 4.5. Reference Standard and Place in the Clinical Pathway

All included studies used reader-assigned labels of CE frames as the reference standard (Section 3.3). The reported sensitivity, specificity, and accuracy therefore quantify agreement between the algorithm and expert CE image interpretation; they do not measure the accuracy of CE itself for identifying the bleeding source. No included study compared AI-assisted CE with conventional flexible endoscopy, device-assisted enteroscopy, surgery, or clinical follow-up. Comparative evidence on the diagnostic role of CE itself comes from clinical studies outside the scope of this review: a meta-analysis showed a higher diagnostic yield of CE than of push enteroscopy and small-bowel radiography in patients with obscure GI bleeding [41], and a multicentre prospective study of a commercially available AI system for reading CE in suspected small-bowel bleeding reported a higher detection rate of clinically relevant lesions and a reduction in reading time from 33.7 to 3.8 min compared with standard reading [42]. Because that study evaluated a commercial system for detecting small-bowel lesions with bleeding potential rather than developing or validating a bleeding-detection model, it did not meet our eligibility criteria; however, it illustrates the patient-level, prospective evidence that is still lacking for the algorithms reviewed here. This is consistent with the clinical role of CE: CE is used mainly for suspected small-bowel bleeding after non-diagnostic upper endoscopy and colonoscopy [13,14], and its findings are confirmed and treated by device-assisted enteroscopy. AI is therefore best regarded as a reading aid within CE, not as an alternative to EGD or colonoscopy. Future studies should define the reference standard at the patient level, for example confirmed small-bowel bleeding source on enteroscopy or clinical follow-up, in addition to frame-level expert labels.

### 4.6. Comparison with the Previous Systematic Review

Singh et al. reviewed 114 studies of ML for VCE-based bleeding analysis published between 2008 and 2024, including studies in which bleeding was one of several abnormality classes, and assessed 104 of them with QUADAS-2 and PROBAST [22]. Twelve of the 15 studies in the present review were also included in that review (Table 1); the remaining three were published between 2023 and 2025 [35,36,37]. The two reviews reach consistent conclusions: Singh et al. reported accuracies often exceeding 95% and found that 88.5% of studies lacked external validation, whereas in the present review the median accuracy was 98.5% and only two of the 15 studies (13.3%) were at low risk for validation. Reported accuracy did not increase over time (median 98.2% for studies published in 2020–2021, *n* = 7, and 98.7% for those published in 2022–2025, *n* = 6), and the only two studies at low risk for validation were published in 2020 and 2021 [18,25]. The most recent studies added new task types, namely attention-based classification and YOLO-based detection [36,37], but neither reported external or patient-level validation. The present review extends the previous findings in two respects. First, Singh et al. reported that internal validation by cross-validation or bootstrapping was used in 93.3% of studies, with only 2.9% at high risk in this respect, whereas our framework additionally required patient-level separation, which no included study reported. Second, our framework explicitly assessed data leakage from augmentation before partitioning, which was identified in four of the 15 studies. Together, these findings indicate that the gap between reported performance and the strength of the supporting evidence has not narrowed. Thus, whereas the previous review maps the broad field of ML for VCE bleeding analysis, the present review provides a focused, bleeding-specific appraisal that readers can use to judge how far current AI systems are from clinical use.

### 4.7. Research Gaps and Future Directions

**Figure 3:**
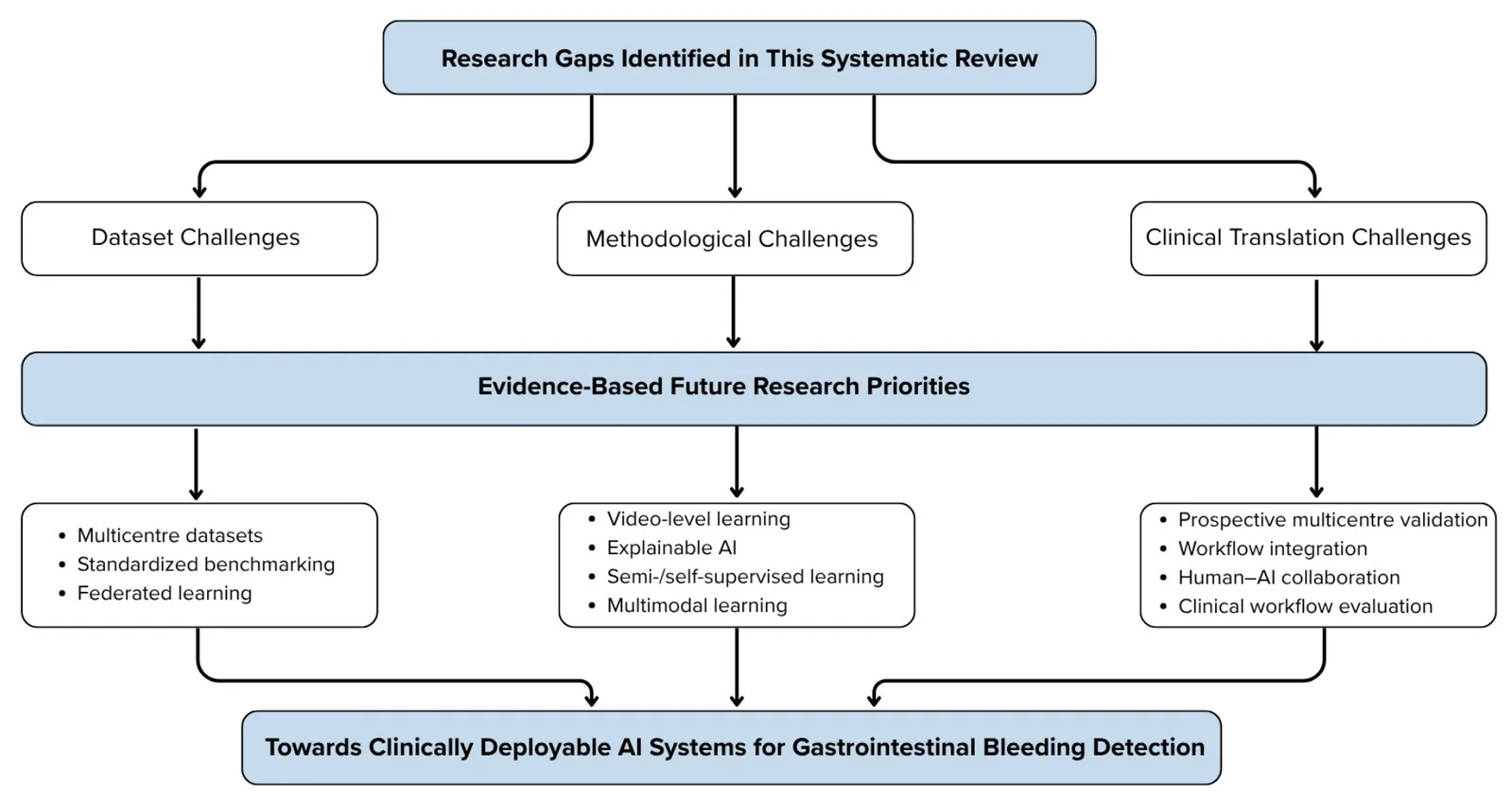
Evidence-based research roadmap derived from this systematic review.

Based on the deficiencies identified in this review, Figure 3 organizes future priorities into three domains: data, methodology, and clinical translation.

With respect to data, the field requires larger, multicentre CE datasets with documented patient provenance, multiple capsule systems, and realistic class distributions, together with standardized benchmarks with fixed patient-level training and test partitions. The included studies used at least seven different datasets [16,18,30,31,35,36,37], which precludes direct comparison. Federated learning may allow multicentre model development without sharing patient data.

With respect to methodology, future studies should report patient-level splits, apply augmentation only to training data, report per-patient and per-examination results, and follow AI-specific reporting guidance. Because CE is a video modality, models that exploit temporal information across consecutive frames, and segmentation or detection models that localize bleeding, are more aligned with clinical reading than frame-level classifiers. Semi-supervised and self-supervised learning may reduce the annotation burden that currently limits localization tasks.

With respect to clinical translation, prospective multicentre studies should evaluate AI systems on complete CE examinations within routine reading workflows, using patient-level reference standards and clinically meaningful outcomes such as reading time, diagnostic yield, missed lesions, and reader confidence.

### 4.8. Limitations of This Review

This review has several limitations. First, the search covered six sources (Google Scholar, PubMed, Scopus, IEEE Xplore, ScienceDirect, and MDPI); studies indexed only in other databases, such as Embase or Web of Science, may have been missed. Database-specific search histories were not prospectively archived, so the exact source-specific syntax could not be reproduced retrospectively, and the review was not prospectively registered. Second, eligibility was restricted to English-language, peer-reviewed journal articles. Conference papers, including those published in proceedings series, and preprints were excluded; because much AI research in this engineering-oriented field is published in conference proceedings, relevant evidence may have been omitted, and the findings may not represent the full body of work. Third, screening, data extraction, and risk-of-bias assessment were each performed by one reviewer and verified in full by a second reviewer, rather than conducted in duplicate by two independent reviewers. Fourth, the AI-adapted risk-of-bias framework, although informed by QUADAS-2 and PROBAST, is not itself a formally validated instrument; it was used because an AI-specific diagnostic-accuracy tool (QUADAS-AI) was not available at the time of assessment and because PROBAST+AI addresses prediction-model rather than image-level algorithm development studies [39,40]. Its ratings should therefore be interpreted as structured judgements rather than validated scores. Fifth, substantial heterogeneity in datasets, tasks, validation designs, and metrics precluded meta-analysis, assessment of publication bias, and formal grading of the certainty of evidence. Sixth, one included study [28] was published in a journal whose coverage in Scopus ended in 2021; it was retained because the journal was indexed at the time of publication, and excluding it would not change the findings of this review (accuracy range unchanged; median accuracy 98.7% instead of 98.5%; validation, data-handling, and clinical-applicability findings unaffected). Finally, performance values were extracted as reported by the original authors and could not be verified independently; in two studies, accuracy values differed between the abstract and the main text [26,34], and the values reported in the main results were used.

## 5. Conclusions

This systematic review of 15 peer-reviewed journal studies shows that AI models for GI bleeding detection in CE consistently report high frame-level accuracy, from 90.6% to 99.9% (median 98.5%), across traditional machine learning, CNN, transfer-learning, attention-based, segmentation, and object-detection approaches. However, high reported performance should not be equated with clinical readiness. No included study separated training and test data at the patient level, four applied data augmentation before partitioning the data, only two performed independent or cross-dataset testing, only four used data from clinical patient examinations, and none evaluated an AI system prospectively, on complete examinations, or against a patient-level reference standard. The lowest accuracies came from the evaluations closest to clinical practice.

The take-home message is that AI achieves high reported accuracy in recognizing bleeding in curated CE images, but its generalizability to new patients, centres, and capsule systems, and its clinical benefit, remain unproven. For clinical practice, the algorithms reviewed here should currently be regarded as research tools that may support, but not replace, expert CE reading. Progress now depends less on new architectures than on better evidence: multicentre datasets with patient-level partitioning, external validation across capsule systems, transparent handling of augmentation and class balance, patient-level reference standards, and prospective studies measuring reading time and diagnostic yield within the clinical pathway, in which CE serves as the principal small-bowel investigation after non-diagnostic upper endoscopy and colonoscopy.

## Figures and Tables

**Table 1:** Comparison of the present review with the recent systematic review by Singh et al. [22].

Aspect	Singh et al. [22]	This Review
Review scope	ML methodologies for VCE-based bleeding analysis	AI approaches exclusively for GI bleeding detection in capsule endoscopy
Study selection	Included bleeding-focused studies together with studies evaluating bleeding alongside other GI abnormalities	Included only peer-reviewed journal studies in which GI bleeding was the primary research objective. Twelve of the 15 included studies [16,18,25,26,27,28,29,30,31,32,33,34] were also included by Singh et al.; three [35,36,37] were not.
Publication period	2008–2024	2020–2025
Included studies	114	15
Quality appraisal	QUADAS-2 and PROBAST applied to 104 studies	AI-adapted risk-of-bias framework (six domains) informed by QUADAS-2 and PROBAST, with explicit rules on data leakage and patient-level separation
Main contribution	Broad overview of ML methodologies for VCE bleeding analysis, including multi-class studies, mapping the wider field	Focused reference on AI for GI bleeding detection: bleeding-specific synthesis with an AI-specific methodological quality assessment, critical comparison of AI approaches, identification of bleeding-specific research gaps, and an evidence-based roadmap for future clinical translation

**Table 2:** Inclusion and exclusion criteria applied during study selection.

Inclusion Criteria	Exclusion Criteria
Published between 2020 and 2025 (date of first online publication).	Published before 2020.
Focused primarily on GI bleeding in CE.	Focused on other GI abnormalities without a primary emphasis on bleeding.
Applied machine learning or deep learning for bleeding classification, detection, localization, or segmentation.	Did not employ AI-based approaches or relied solely on conventional image processing or statistical methods.
Original peer-reviewed journal articles in English.	Review articles, editorials, book chapters, theses, conference papers (including proceedings series such as *Procedia Computer Science*) and abstracts, posters, patents, and preprints.
Reported sufficient methodological details, dataset information, validation strategy, and quantitative performance metrics.	Insufficient methodological transparency, incomplete experimental reporting, or inadequate data for meaningful comparison.

**Table 3:** AI-adapted methodological quality assessment framework used to evaluate the included studies.

Domain	Assessment Criterion
Dataset Representativeness	Clinically representative CE dataset with clearly described dataset source and patient selection.
Reference Standard	Expert-annotated bleeding labels or validated ground-truth reference.
Validation Strategy	Independence of test data: patient-level splitting, cross-dataset testing, or external validation on an independent CE dataset.
Data Handling	Clear reporting of preprocessing, data augmentation, class imbalance management, and data partitioning, with augmentation confined to training data.
Reporting Transparency	Sufficient methodological details to ensure reproducibility, including model architecture, training procedures, and evaluation metrics.
Clinical Applicability	Evaluation on CE data from clinical patient examinations, supporting translation into routine CE interpretation.

**Table 4:** Characteristics of the studies included in the systematic review, grouped by primary AI category. NR: not reported; CV: cross-validation.

Ref.	Year	AI Approach	Task	Dataset	Sample Size	Validation
*Traditional machine learning*						
[26]	2021	Traditional ML (fuzzy logic + PCA + quadratic SVM)	Classification	Charisis et al. WCE	2393 images	10-fold CV
[32]	2021	Traditional ML (GMM superpixels + SVM)	Detection + segmentation	Two standard WCE datasets + GrowCut	3294 images; 100 segmentation images	50:50 split + 10-fold CV
[34]	2023	Traditional ML (color- and texture-based)	Classification + localization	Public WCE videos (source not specified)	2300 images (30 videos)	10-fold CV
*CNN-based deep learning (trained end-to-end)*						
[25]	2020	CNN ensemble	Classification	Public (training) + real clinical video (independent test)	3895 public + 5000 real video images	85:15 split (10 trials); independent real-video test
[28]	2021	CNN	Classification	“Kvasir” + private WCE video	4778 images (+1500 video frames; role unclear)	70:30 split
[30]	2024	Custom CNN	Classification	Kvasir-Capsule	892 images	70:30; 80:20 split; 56-frame video test
*Transfer learning/feature fusion*						
[18]	2021	Pretrained VGG19 + InceptionV3 + ResNet50 features + SVM	Classification	KID + MICCAI 2017	2352 + 3895 original images (augmented to 56,448 + 93,480)	10-fold CV; cross-dataset
[31]	2021	Transfer learning (AlexNet) + SegNet	Classification + segmentation	CapsuleEndoscopy.org + KID	2350 frames (32 videos); 335 KID images for segmentation	Random 60:40 split, repeated 10 times
[27]	2021	Transfer learning (MobileNet + custom CNN)	Classification	Private (33 patients) + internet-collected WCE	5000 images	Split (1500/150) + 10-fold CV; internet-collected test set
[16]	2022	Transfer learning (Xception)	Classification	Single-centre CE (1483 examinations)	22,095 images	Random 80:20 split
[35]	2023	Transfer learning (InceptionV3)	Classification	Real clinical CE (39 patients)	13,116 images	Random finding-level hold-out
*Segmentation network*						
[33]	2022	DFCA-Net	Segmentation	Kvasir-Capsule + Kvasir	568 images; augmented to 2272	70/10/20 split
*Attention-based/hybrid architectures*						
[29]	2023	CNN-GRU	Classification	MICCAI 2017	3295 images	60/20/20 split + 5-fold CV
[37]	2025	Attention-enhanced residual U-Net (HARU-Net)	Classification	Kaggle GI bleeding	226 original images augmented to 4746	5-fold CV
*Object detection*						
[36]	2025	YOLOv11 object detection	Detection	Auto-WCEBleedGen V2	2618 images	NR

**Table 5:** Performance metrics reported by the included studies. NR: Not Reported.

Ref.	Year	Acc. (%)	Sens. (%)	Spec. (%)	Prec. (%)	F1 (%)	Other Evaluation Metrics
[26]	2021	98.20	98.00	98.00	93.00	95.40	NPV = 99.00%
[32]	2021	99.88	99.83	100.00	99.83	99.91	AUC = 1.00; Dice = 99.28–99.05%; IoU = 98.58–98.13%
[34]	2023	98.86	95.20	98.32	88.38	NR	AUC = 0.9771; abstract reports 95.75% accuracy
[25]	2020	95.00	92.00	96.00	91.00	92.00	Independent real video: Acc. = 93%
[28]	2021	95.70	97.10	94.60	NR	NR	NR
[30]	2024	99.44	99.41	NR	99.47	99.44	70:30 split: Acc. = 98.51%
[18]	2021	95.70	95.82	95.53	97.04	96.43	KID: Acc. = 98.20%; cross-dataset (MICCAI→KID): Acc. = 93.41%
[31]	2021	99.44	97.51	99.88	99.50	98.49	Weighted IoU = 90.69%; Global Acc. = 94.42%; KID: Acc. = 83.24%
[27]	2021	99.30	99.40	NR	100.00	99.70	Internet-collected set: Acc. = 97.8%
[16]	2022	98.50	98.60	98.90	98.70	NR	AUC = 1.00
[35]	2023	90.60	89.40	91.70	90.90	NR	NPV = 90.30%
[33]	2022	NR	NR	NR	NR	NR	Pixel Acc. = 91.26%; mIoU = 86.86%; MPA = 95.21%
[29]	2023	99.39	99.09	99.54	99.09	99.09	AUC = 1.00; 5-fold CV: Acc. = 98.33%
[37]	2025	98.21	98.02	NR	98.34	98.21	NR
[36]	2025	NR	69.15	NR	NR	NR	AP = 82.40%; mAP@0.5 = 78.98%; mAP@0.5:0.95 = 48.31%

**Table 6:** Methodological quality assessment of the included studies using the proposed AI-adapted risk-of-bias framework (revised ratings). L = Low risk; H = High risk; U = Unclear risk. Cell shading indicates the rating: green, low risk; yellow, unclear risk; red, high risk.

Study	Dataset	Reference	Validation	Data	Reporting	Clinical
	Rep.	Std.	Strategy	Handling	Transp.	Applic.
Al Mamun et al. [26]	**U**	**U**	**U**	**L**	**L**	**U**
Rathnamala and Jenicka [32]	**L**	**L**	**U**	**L**	**L**	**U**
Vajravelu et al. [34]	**U**	**U**	**U**	**L**	**U**	**U**
Pannu et al. [25]	**L**	**L**	**L**	**L**	**L**	**L**
Latha et al. [28]	**U**	**U**	**H**	**U**	**U**	**U**
Farej et al. [30]	**L**	**L**	**H**	**L**	**L**	**U**
Caroppo et al. [18]	**L**	**L**	**L**	**H**	**L**	**U**
Ghosh and Chakareski [31]	**L**	**L**	**H**	**L**	**L**	**U**
Rustam et al. [27]	**L**	**L**	**U**	**H**	**L**	**L**
Saraiva et al. [16]	**L**	**L**	**H**	**L**	**L**	**L**
Turck et al. [35]	**L**	**L**	**H**	**L**	**L**	**L**
Li et al. [33]	**L**	**L**	**H**	**H**	**L**	**U**
Lafraxo et al. [29]	**L**	**L**	**U**	**L**	**L**	**U**
Balasubaramanian et al. [37]	**H**	**U**	**U**	**H**	**L**	**H**
Chai and Zhang [36]	**U**	**L**	**U**	**U**	**U**	**U**

**Table 7:** Comparative synthesis of artificial intelligence approaches for gastrointestinal bleeding detection in capsule endoscopy.

AI Approach	*n*	Included Studies	Reported Performance Range	Recurring Limitations
Traditional Machine Learning	3	[26,32,34]	Accuracy: 98.2–99.9%	Handcrafted features; image-level splits or cross-validation; no external validation.
CNN-based Deep Learning (end-to-end)	3	[25,28,30]	Accuracy: 95.0–99.4%	Frame-level analysis; small or incompletely described datasets; one independent clinical-video test [25].
Transfer Learning/Feature Fusion	5	[16,18,27,31,35]	Accuracy: 90.6–99.4%	Random image- or finding-level splits; single-centre data; one cross-dataset evaluation [18].
Segmentation Network	1	[33]	mIoU: 86.9%	Pixel-level annotation required; augmentation before partitioning; no external validation.
Attention/Hybrid Architectures	2	[29,37]	Accuracy: 98.2–99.4%	Architectural complexity; small or heavily augmented datasets; no independent validation.
Object Detection	1	[36]	mAP@0.5: 79.0%	Single benchmark dataset; partitioning not reported; no clinical validation.

## Data Availability

No new data were created or generated in this study. All data supporting the findings of this systematic review are available in the cited published literature and in the Supplementary Materials.

## Supplementary Materials

The following supporting information can be downloaded at https://www.mdpi.com/article/10.3390/diagnostics16213449/s1, Table S1: Reports excluded following full-text assessment and reasons for exclusion; Table S2: PRISMA 2020 checklist; Table S3: Changes to risk-of-bias ratings between the initial and revised assessments, with justification. References [31,37,38,43,44,45,46,47,48,49] are cited in the Supplementary Materials.

## Abbreviations

The following abbreviations are used in this manuscript:

AI	Artificial Intelligence
AUC	Area Under the Curve
CE	Capsule Endoscopy
CNN	Convolutional Neural Network
CV	Cross-Validation
DL	Deep Learning
EGD	Esophagogastroduodenoscopy
GI	Gastrointestinal
GRU	Gated Recurrent Unit
IoU	Intersection over Union
KNN	k-Nearest Neighbors
mAP	Mean Average Precision
mIoU	Mean Intersection over Union
ML	Machine Learning
NPV	Negative Predictive Value
OGD	Oesophagogastroduodenoscopy
PCA	Principal Component Analysis
PRISMA	Preferred Reporting Items for Systematic Reviews and Meta-Analyses
PROBAST	Prediction model Risk Of Bias ASsessment Tool
QUADAS	Quality Assessment of Diagnostic Accuracy Studies
SVM	Support Vector Machine
VCE	Video Capsule Endoscopy
WCE	Wireless Capsule Endoscopy
