# Supplementary material for: Artificial Intelligence Approaches for Gastrointestinal Bleeding Detection in Capsule Endoscopy: A Systematic Review and Critical Analysis
Source: Diagnostics (Basel). 2026 Oct 22;16(21):3449. doi: 10.3390/diagnostics16213449 (PMC13761944; doi:10.3390/diagnostics16213449)
Supplement: Supplementary file 1 [file diagnostics-16-03449-s001.zip › diagnostics-4550025-supplementary.pdf]

Supplementary Materials

Manuscript Title: Artificial Intelligence Approaches for Gastrointestinal Bleeding Detection in Capsule Endoscopy: A Systematic Review and Critical Analysis

Table S1. Reports excluded following full-text assessment and reasons for exclusion ($n = 9$).

**Excluded Report**	**Reason for Exclusion**
Insufficient methodological reporting ($n = 2$)	
Automated Small Intestine Bleeding Detection Using Wireless Capsule Endoscopy Images [1]	Insufficient methodological reporting; the dataset description, train–test split, validation protocol, and performance evaluation methodology were insufficiently described.
GI Bleeding Detection in WCE Images Using E-ORB and ME-DEEP CAPSNET [2]	Insufficient methodological transparency; dataset characteristics, sample size, validation protocol, and experimental setup were not adequately reported.
Conference proceedings papers ($n = 3$)	
ColonNet: A Hybrid of DenseNet121 & U-Net Models for Detection and Segmentation of GI Bleeding [4]	Conference proceeding; excluded because only original peer-reviewed journal articles were eligible for inclusion.
Semi-supervised Learning for Segmentation of Bleeding Regions in Video Capsule Endoscopy [5]	Conference proceedings paper (International Conference on Biomimetic Intelligence and Robotics, published in *Procedia Computer Science*); excluded because only original peer-reviewed journal articles were eligible for inclusion.
Beyond X-ray: Deep Learning Solutions for Gastrointestinal Bleeding [6]	Conference proceedings paper (Sixth International Conference on Futuristic Trends in Networks and Computing Technologies, FTNCT06, published in *Procedia Computer Science*); excluded because only original peer-reviewed journal articles were eligible for inclusion.
Preprint, not peer-reviewed ($n = 1$)	
Automated Bleeding Detection and Classification in Wireless Capsule Endoscopy with YOLOv8-X [3]	Not peer-reviewed; available as an arXiv challenge submission.
Duplicate reports ($n = 2$)	
Attention-Enhanced Residual U-Net with Histogram Equalization for Automated Classification of Gastrointestinal Bleeding Disorders [7]	Duplicate report of a study already identified during the screening process.
Deep Transfer Learning for Automated Intestinal Bleeding Detection [8]	Duplicate report of a study already identified during the screening process.
Did not meet the eligibility criteria ($n = 1$)	
Gastrointestinal Bleeding Classification in Capsule Endoscopy Images using Deep Learning [9]	Did not meet the predefined eligibility criteria; the positive class was not restricted to bleeding and included mixed lesion categories, including active bleeding, clots, and ulcers. The report was also a conference publication.

References

1. Ajay, A.; Selvaperumal, S.K. Automated Small Intestine Bleeding Detection Using Wireless Capsule Endoscopy Images. 2021. Available online: `https://api.semanticscholar.org/CorpusID:235374666` (accessed on 21 October 2026).

2. Rathnamala, S.; Rajagopal, A.; Arunachalam, M.; Dhanasekaran, V. GI Bleeding Detection in WCE Images Using E-ORB and ME-DEEP CAPSNET. *Int. J. Environ. Sci.* **2025**, *11*, 147–160. https://doi.org/10.64252/358k2389.
3. Shekar, P.C.; Kanhangad, V.; Maheshwari, S.; Kumar, T.S. Automated Bleeding Detection and Classification in Wireless Capsule Endoscopy with YOLOv8-X. *arXiv* **2024**, arXiv:2412.16624. https://doi.org/10.48550/arXiv.2412.16624.
4. Singh, A.; Prakash, S.; Das, A.; Kushwaha, N.; Singh, B. ColonNet: A Hybrid of DenseNet121 & U-Net Models for Detection and Segmentation of GI Bleeding. In Proceedings of the 2025 IEEE International Conference on Interdisciplinary Approaches in Technology and Management for Social Innovation (IATMSI), 2025, Volume 3, pp. 1–5. https://doi.org/10.1109/IATMSI64286.2025.10984586.
5. Li, H.; Wu, Y.; Bai, L.; Wang, A.; Chen, T.; Ren, H. Semi-supervised Learning for Segmentation of Bleeding Regions in Video Capsule Endoscopy. In Proceedings of the International Conference on Biomimetic Intelligence and Robotics; *Procedia Comput. Sci.* **2023**, *226*, 29–35. https://doi.org/10.1016/j.procs.2023.10.632.
6. Kumar, S.Y.; J M, A.G.; Vimal, T.; Abhishek, S.; T, A. Beyond X-ray: Deep Learning Solutions for Gastrointestinal Bleeding. In Proceedings of the Sixth International Conference on Futuristic Trends in Networks and Computing Technologies (FTNCT06); *Procedia Comput. Sci.* **2025**, *259*, 1306–1315. https://doi.org/10.1016/j.procs.2025.04.085.
7. Balasubaramanian, S.; Shyamala Devi, M.; Kavitha, K.; Balasubramaniam, S. Attention-Enhanced Residual U-Net with Histogram Equalization for Automated Classification of Gastrointestinal Bleeding Disorders (duplicate record). https://doi.org/10.1016/j.bspc.2025.108371.
8. Ghosh, T.; Chakareski, J. Deep Transfer Learning for Automated Intestinal Bleeding Detection in Capsule Endoscopy Imaging (duplicate record). *J. Digit. Imaging* **2021**, *34*, 404–417. https://doi.org/10.1007/s10278-021-00428-3.
9. Asha Rani, K.P.; Gowrishankar, S.; Amrutesh, A. Gastrointestinal Bleeding Classification in Capsule Endoscopy Images using Deep Learning. In Proceedings of the 2024 4th International Conference on Data Engineering and Communication Systems (ICDECS), 2024, pp. 1–6. https://doi.org/10.1109/ICDECS59733.2023.10502983.

Supplementary Table S2. PRISMA 2020 Checklist

Artificial Intelligence Approaches for Gastrointestinal Bleeding Detection in Capsule Endoscopy: A Systematic Review and Critical Analysis

Page and section numbers refer to the revised manuscript (Revision 1).

Section	Topic	Item #	Checklist item	Location where item is reported
**TITLE**	Title	1	Identify the report as a systematic review.	p. 1, Title.
**ABSTRACT**	Abstract	2	See the PRISMA 2020 for Abstracts checklist.	p. 1, Abstract (15 included studies; accuracy range and median; principal risk-of-bias findings).
**INTRODUCTION**	Rationale	3	Describe the rationale for the review in the context of existing knowledge.	pp. 1–3, Introduction (clinical pathway and role of capsule endoscopy); Table 1 (comparison with Singh et al., exact overlap of 8 of 15 studies), p. 3; Section 4.6, p. 18.
	Objectives	4	Provide an explicit statement of the objective(s) or question(s) the review addresses.	p. 3, final paragraph of the Introduction; p. 3, Section 2.1 Review Design.
**METHODS**	Eligibility criteria	5	Specify the inclusion and exclusion criteria for the review and how studies were grouped for the syntheses.	pp. 4–5, Section 2.4 Eligibility Criteria and Table 2 (journal articles only; proceedings series classified as conference papers; date of first online publication); p. 9, Section 2.8 (one primary AI category per study).
	Information sources	6	Specify all databases, registers, websites, organisations, reference lists and other sources searched or consulted to identify studies. Specify the date when each source was last searched or consulted.	p. 4, Section 2.2 Information Sources (Google Scholar, PubMed, Scopus, IEEE Xplore, ScienceDirect, MDPI; citation searching; each last searched June 2026).
	Search strategy	7	Present the full search strategies for all databases, registers and websites, including any filters and limits used.	p. 4, Section 2.3 Literature Search Strategy (common Boolean strategy, English language, 2020–2025). Exact source-specific historical syntax unavailable and transparently stated; limitation noted in Section 4.8, pp. 19–20.
	Selection process	8	Specify the methods used to decide whether a study met the inclusion criteria of the review, including how many reviewers screened each record and each report retrieved, whether they worked independently, and if applicable, details of automation tools used in the process.	p. 5, Section 2.5 Study Selection (one reviewer with all decisions checked by a second reviewer, not in duplicate; disagreements resolved by consensus, no automation tools).
	Data collection process	9	Specify the methods used to collect data from reports, including how many reviewers collected data from each report, whether they worked independently, any processes for obtaining or confirming data from study investigators, and if applicable, details of automation tools used in the process.	p. 6, Section 2.6 Data Collection Process and Data Items (one reviewer with all extracted data checked by a second reviewer against the original publications, not in duplicate; no automation tools).
	Data items	10a	List and define all outcomes for which data were sought. Specify whether all results that were compatible with each outcome domain in each study were sought, and if not, the methods used	p. 6, Section 2.6 (accuracy, sensitivity/recall, specificity, precision, F1, NPV, AUC, Dice, IoU, global accuracy, mAP and other task-specific metrics; rule for selecting the headline

Section	Topic	Item #	Checklist item	Location where item is reported
			to decide which results to collect.	configuration; additional results reported in Table 5).
	Data items	10b	List and define all other variables for which data were sought. Describe any assumptions made about any missing or unclear information.	p. 6, Section 2.6 (publication year, approach, task, dataset, sample size, reference standard, augmentation, validation, computational resources, findings, limitations; missing metrics recorded as NR and not imputed).
	Study risk of bias assessment	11	Specify the methods used to assess risk of bias in the included studies, including details of the tool(s) used, how many reviewers assessed each study and whether they worked independently, and if applicable, details of automation tools used in the process.	pp. 7–9, Sections 2.7 and 2.7.1 and Table 3 (AI-adapted framework informed by QUADAS-2 and PROBAST; six predefined domains; explicit operational rating rules; one reviewer with verification of all ratings by a second reviewer and disagreements resolved by consensus; no automation tools; rationale for not using QUADAS-AI/PROBAST+AI).
	Effect measures	12	Specify for each outcome the effect measure(s) used in the synthesis or presentation of results.	Not applicable to pooled effect estimates because no meta-analysis was undertaken. Diagnostic performance measures are listed in Section 2.6, p. 6, and reported in Table 5, p. 11.
	Synthesis methods	13a	Describe the processes used to decide which studies were eligible for each synthesis.	p. 9, Section 2.8 (each study assigned to one primary methodological category, used consistently in Tables 4 and 7 and the Results).
	Synthesis methods	13b	Describe any methods required to prepare the data for presentation or synthesis, such as handling of missing summary statistics, or data conversions.	p. 6, Section 2.6, and p. 9, Section 2.8 (values retained as published; missing outcomes designated NR; no imputation).
	Synthesis methods	13c	Describe any methods used to tabulate or visually display results of individual studies and syntheses.	p. 9, Section 2.8; Tables 4–7 and Figures 2–3.
	Synthesis methods	13d	Describe any methods used to synthesize results and provide a rationale for the choice(s). If meta-analysis was performed, describe the model(s), method(s) to identify the presence and extent of statistical heterogeneity, and software package(s) used.	p. 9, Section 2.8 (qualitative narrative synthesis; meta-analysis not performed because of substantial methodological and clinical heterogeneity).
	Synthesis methods	13e	Describe any methods used to explore possible causes of heterogeneity among study results (e.g. subgroup analysis, meta-regression).	p. 9, Section 2.8 (descriptive comparison of datasets, sample sizes, AI approaches, task types, validation strategies and performance metrics).
	Synthesis methods	13f	Describe any sensitivity analyses conducted to assess robustness of the synthesized results.	p. 9, Section 2.8. No formal sensitivity analysis was conducted because no pooled quantitative synthesis was undertaken; the effect of excluding one study published in a journal whose indexing status changed was checked descriptively (Section 4.8, pp. 19–20).

Section	Topic	Item #	Checklist item	Location where item is reported
	Reporting bias assessment	14	Describe any methods used to assess risk of bias due to missing results in a synthesis (arising from reporting biases).	p. 9, Section 2.8. Formal statistical assessment was not performed because no meta-analysis was undertaken; reporting limitations were considered qualitatively through the reporting-transparency domain.
	Certainty assessment	15	Describe any methods used to assess certainty (or confidence) in the body of evidence for an outcome.	p. 9, Section 2.8. GRADE was not applied; strength and applicability were considered through the methodological quality assessment and critical qualitative synthesis.
**RESULTS**	Study selection	16a	Describe the results of the search and selection process, from the number of records identified in the search to the number of studies included in the review, ideally using a flow diagram.	p. 6, Figure 1 (PRISMA 2020 flow diagram); p. 5, Section 2.5; p. 9, Section 3.1 (218 records; 24 reports assessed; 9 excluded; 15 studies included).
	Study selection	16b	Cite studies that might appear to meet the inclusion criteria, but which were excluded, and explain why they were excluded.	p. 5, Section 2.5; p. 9, Section 3.1; Supplementary Table S1 (nine full-text-excluded reports individually cited with reasons, including three conference proceedings papers).
	Study characteristics	17	Cite each included study and present its characteristics.	p. 10, Table 4 (grouped by primary AI category); Sections 3.4–3.9, pp. 11–14.
	Risk of bias in studies	18	Present assessments of risk of bias for each included study.	p. 15, Table 6, Figure 2 and Section 3.10.
	Results of individual studies	19	For all outcomes, present, for each study: (a) summary statistics for each group (where appropriate) and (b) an effect estimate and its precision (e.g. confidence/credible interval), ideally using structured tables or plots.	p. 11, Table 5; Sections 3.4–3.9, pp. 11–14. Precision estimates were generally not reported by the source studies; the review presents the reported diagnostic performance metrics.
	Results of syntheses	20a	For each synthesis, briefly summarise the characteristics and risk of bias among contributing studies.	Sections 3.2–3.10, pp. 9–15; Table 7 and Section 4.3, pp. 16–17.
	Results of syntheses	20b	Present results of all statistical syntheses conducted. If meta-analysis was done, present for each the summary estimate and its precision (e.g. confidence/credible interval) and measures of statistical heterogeneity. If comparing groups, describe the direction of the effect.	Not applicable. No statistical meta-analysis or pooled quantitative synthesis was conducted (Section 2.8, p. 9).
	Results of syntheses	20c	Present results of all investigations of possible causes of heterogeneity among study results.	Descriptive heterogeneity findings in Sections 4.3 and 4.4, pp. 17–18 (dataset size and composition, augmentation, validation strategy, task definition and evaluation differences).
	Results of syntheses	20d	Present results of all sensitivity analyses conducted to assess the robustness of the synthesized results.	No formal sensitivity analyses were conducted because no pooled quantitative synthesis was undertaken (Section 2.8, p. 9). A descriptive check showed that excluding one study published in a journal whose indexing status changed would not alter the findings (Section 4.8, pp. 19–20).

Section	Topic	Item #	Checklist item	Location where item is reported
	Reporting biases	21	Present assessments of risk of bias due to missing results (arising from reporting biases) for each synthesis assessed.	Not applicable as a formal synthesis-level assessment. Formal publication or selective-reporting bias tests were not performed; reporting limitations are described in Section 2.8, p. 9, and reflected in the reporting-transparency domain (Table 6).
	Certainty of evidence	22	Present assessments of certainty (or confidence) in the body of evidence for each outcome assessed.	Not applicable as a formal GRADE assessment. Rationale in Section 2.8, p. 9; confidence and applicability discussed in Sections 4.1 and 4.4, pp. 16–18.
**DISCUSSION**	Discussion	23a	Provide a general interpretation of the results in the context of other evidence.	Sections 4.1–4.6, pp. 16–19 (including comparison with Singh et al., Section 4.6).
	Discussion	23b	Discuss any limitations of the evidence included in the review.	Section 3.10, p. 15; Sections 4.4 and 4.5, pp. 17–18 (validation design, data leakage, dataset representativeness, reference standards, clinical applicability).
	Discussion	23c	Discuss any limitations of the review processes used.	pp. 19–20, Section 4.8 Limitations of This Review.
	Discussion	23d	Discuss implications of the results for practice, policy, and future research.	Sections 4.5 and 4.7, pp. 18–19 (Figure 3); Conclusions, p. 20.
**OTHER INFORMATION**	Registration and protocol	24a	Provide registration information for the review, including register name and registration number, or state that the review was not registered.	p. 3, Section 2.1 Review Design: the review was not registered (e.g., PROSPERO).
	Registration and protocol	24b	Indicate where the review protocol can be accessed, or state that a protocol was not prepared.	p. 3, Section 2.1 Review Design: no prospectively registered or published review protocol was available.
	Registration and protocol	24c	Describe and explain any amendments to information provided at registration or in the protocol.	p. 3, Section 2.1 Review Design: amendments to a registered protocol were not applicable.
	Support	25	Describe sources of either financial or non-financial support for the review, and the role of the funders or sponsors in the review.	p. 20, Funding: no external funding.
	Competing interests	26	Declare any competing interests of review authors.	p. 20, Conflicts of Interest: the authors declare no conflicts of interest.
	Availability of data, code and other materials	27	Report which of the following are publicly available and where they can be found: template data collection forms; data extracted from included studies; data used for all analyses; analytic code; any other materials used in the review.	p. 20, Data Availability Statement: no new data were created; data supporting the review are available in the cited published literature and the Supplementary Materials (Table S1; Table S2).

From: Page MJ, McKenzie JE, Bossuyt PM, Boutron I, Hoffmann TC, Mulrow CD, et al. The PRISMA 2020 statement: an updated guideline for reporting systematic reviews. BMJ 2021;372:n71. doi: 10.1136/bmj.n71.

Table S3. Changes to risk-of-bias ratings between the initial and revised assessments, with justification. During revision, the rating criteria were made operational for three recurring situations (studies reporting both a random split and k-fold cross-validation, the definition of external validation, and the evidence required for clinical applicability), and all 90 ratings (15 studies $\times$ 6 domains) were re-assessed against the full-text publications by one reviewer and verified by a second reviewer. In total, 63 ratings (70%) were unchanged and 27 (30%) changed; only the changed ratings are listed. Reports excluded during revision (conference proceedings papers) are not shown.

Study	Domain	Initial	Revised	Justification (operational rule applied)
Pannu et al. (2020)	Validation strategy	High	Low	Model tested on an independent, gastroenterologist-annotated clinical CE video (5,000 frames).
	Clinical applicability	Unclear	Low	Evaluated on real clinical CE video.
Al Mamun et al. (2021)	Reference standard	Low	Unclear	Annotation process for the 2,393 images not described.
	Validation strategy	Low	Unclear	10-fold image-level cross-validation without patient-level separation.
Caroppo et al. (2021)	Data handling	Low	High	Augmentation (24-fold) applied to the full dataset before 10-fold cross-validation.
Ghosh and Chakareski (2021)	Validation strategy	Low	High	Repeated random 60/40 image-level split; KID images evaluated separately rather than as a cross-dataset test.
Rustam et al. (2021)	Dataset representativeness	High	Low	5,000 images from 33 patients at a clinical centre, with clinician labels.
	Validation strategy	High	Unclear	Split plus 10-fold cross-validation without patient-level separation; internet-collected test set not counted as external CE validation.
	Data handling	Low	High	Cross-validation performed on augmented data.
	Clinical applicability	Unclear	Low	Evaluated on CE data from a clinical patient cohort.
Latha et al. (2021)	Reference standard	Low	Unclear	Labelling process not described.
	Data handling	Low	Unclear	Role of the additional video frames and data partitioning not clearly reported.
Saraiva et al. (2022)	Clinical applicability	Unclear	Low	Evaluated on CE examinations from a single-centre clinical patient cohort.
Li et al. (2022)	Validation strategy	Unclear	High	Random 7:1:2 image-level split; additional test on conventional endoscopy images not counted as CE external validation.
	Data handling	Low	High	Augmentation applied before data partitioning.
Turck et al. (2023)	Clinical applicability	Unclear	Low	Evaluated on real-world clinical CE examinations annotated by capsule experts.
Vajravelu et al. (2023)	Reporting transparency	Low	Unclear	Source of the video dataset not named; accuracy differs between abstract and main text.
	Clinical applicability	High	Unclear	Evaluated on public CE video images; not a small, heavily augmented image set.
Lafraxo et al. (2023)	Dataset representativeness	Unclear	Low	Validated public CE dataset (MICCAI 2017).
	Validation strategy	High	Unclear	Split plus 5-fold cross-validation without patient-level separation.

Study	Domain	Initial	Revised	Justification (operational rule applied)
Farej et al. (2024)	Dataset representativeness	Unclear	Low	Validated public CE dataset (Kvasir-Capsule).
Chai and Zhang (2025)	Validation strategy	High	Unclear	Data partitioning not reported.
	Data handling	Low	Unclear	Data partitioning and class handling not reported.
	Reporting transparency	Low	Unclear	Key experimental details, including data partitioning, not reported.
Balasubaramanian et al. (2025)	Validation strategy	High	Unclear	5-fold cross-validation without patient-level separation.
	Data handling	Low	High	226 original images augmented approximately 21-fold before data partitioning.
	Clinical applicability	Unclear	High	Evaluation confined to a very small, heavily augmented image set.
